# Hydrogen-Induced Cracking Caused by Galvanic Corrosion of Steel Weld in a Sour Environment

**DOI:** 10.3390/ma14185282

**Published:** 2021-09-14

**Authors:** Jin Sung Park, Jin Woo Lee, Sung Jin Kim

**Affiliations:** 1 Department of Advanced Materials Engineering, Sunchon National University Jungang-ro, Suncheon 540-742, Korea; pjs1352@naver.com; 2 POSCO Technical Research Laboratories, Pohang 790-704, Korea; sjhrte1156@nate.com

**Keywords:** ASTM A516-65 steel, weld, hydrogen-induced cracking, galvanic corrosion, sour environment

## Abstract

This study examined the hydrogen-induced cracking (HIC) caused by galvanic corrosion of an ASTM A516-65 steel weld in a wet sour environment using a combination of standard immersion corrosion test, electrochemical analyses, and morphological observation of corrosion damage. This study showed that the weld metal has lower open circuit potential, and higher anodic and cathodic reaction rates than the base metal. The preferential dissolution and much higher density of localized corrosion damage were observed in the weld metal of the welded steel. On the other hand, the presence of weldment can make steel more susceptible to HIC, specifically, in areas of the base metal but not in the weld metal or heat affected zone, which is in contrast to typical expectations based on metallurgical knowledge. This can be explained by galvanic corrosion interactions between the weldment and the base metal, acting as a small anode and a large cathode, respectively. This type of galvanic couple can provide large surface areas for infusing cathodically-reduced hydrogen on the base metal in wet sour environments, increasing the susceptibility of welded steel to HIC.

## 1. Introduction

Hydrogen degradation of ferrous alloys has attracted considerable attention in the scientific and engineering community for more than 100 years [1,2,3]. Among the degradation phenomena, hydrogen-induced cracking (HIC) and sulfide stress corrosion cracking (SSCC) are major technical problems that remain to be addressed in the petrochemical industries. Premature failure caused by hydrogen embrittlement (HE) occurs mainly at the welded joints of the steel structures [4,5,6]. This is because welds are formed under a welding thermal cycle with rapid heating and cooling processes, and they are normally comprised of dendritic and heterogeneous structures with several metallurgical defects [7,8]. Hence, the welded joint is considered the most critical and problematic part of steel structures.

Sour corrosion is the deterioration that occurs on a metal surface in a highly acidic environment containing H_2_S. This type of corrosion is expected to occur preferentially at the welds when the welded steel structures are exposed to wet sour environments. The sour corrosion process can be briefly summarized as anodic metal dissolution (M → M^n+^ + e^−^) followed by the infusion of cathodically-reduced hydrogen (H^+^ + e^−^ → H) in steel [9,10,11]. The poisoning effect by H_2_S facilitates the absorption of atomic hydrogen, and the hydrogen could be trapped at certain metallurgical defects in steel [9,12,13]. According to internal pressure theory [14], which has been widely accepted as a mechanism of HE in steels, the continuous trapping of atomic hydrogens tends to recombine into molecular hydrogen (H + H → H_2_) and leads to significant volume expansion, resulting in the nucleation of cracks.

The heat affected zone (HAZ) with a coarse grain size can be the most inferior part of the welded steel, and numerous papers have reported the (hydrogen-induced) mechanical degradation of the HAZ depending on the welding heat input [15,16]. In the case of weld metal (WM), welding consumables can be one of the factors controlling the resistance to hydrogen-assisted cracking (HAC) failures. On the other hand the welding consumable is adopted considering mostly the mechanical properties of the base metal (BM). This welding consumable does not guarantee the WM will have high resistance to corrosion or corrosion-induced HAC. In this regard, Pagotto et al. [17] reported a much higher anodic dissolution current at welds relative to the BM of carbon steel in a neutral aqueous environment, employing a scanning vibrating electrode technique (SVET).

In contrast to the common metallurgical aspects described above, the authors found that the HIC ratio of the BM was higher than that of the WM when the welded steel was exposed to a wet sour environment. Moreover, the cracking problem of the BM became worse in the presence of the WM compared to the unwelded steel sample. This is closely associated with the formation of a galvanic couple with the WM and BM in an acidic aqueous environment. In this study, the National Association of Corrosion Engineers (NACE) standard HIC test, diffusible hydrogen measurement, and several electrochemical evaluations were conducted to clarify the mechanistic reason for the more serious damage by HIC in the BM, which can be protected galvanically.

## 2. Experimental Procedure

The test materials under investigation were equivalent to an ASTM A516-65 grade pressure vessel steel plate with a 15 mm thickness produced by an industrial rolling process. The chemical compositions of the two types of steel samples used in this study, termed Steel A and B, are listed in Table 1. The steels were normalized by heating to 910 °C for 8 min and cooled to room temperature in air.

To produce the welded samples for Steel A and B, a double X-groove was produced, and the tandem submerged arc welding (SAW) was performed using two solid wires (OE-SD3, 0.07% C-0.9% Mn-0.3% Si) with diameters of 4 mm each and an OP121TT (0.07% C-1.6% Mn-0.3% Si) flux. The total heat input from the sum of two electrodes was approximately 30 kJ/cm, which was calculated using the welding parameters; these are listed in Table 2.

**Figure 1:**
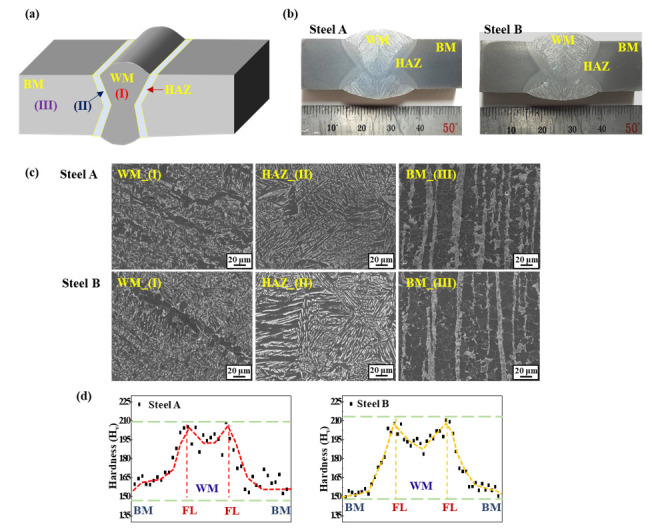
(**a**) Brief schematic diagram of the double-pass welded sample; (**b**) Cross-section view of the two welded samples; (**c**) Microstructures in the WM, HAZ, and BM of the two welded samples; (**d**) Vickers hardness profile of the two welded samples.

A brief schematic diagram of the double-pass welded sample is presented in Figure 1a.

Metallographic observations of the WM, HAZ, and BM by optical microscopy (OM) (Zeiss, Jena, Germany) and field-emission scanning electron microscopy (FE-SEM) (Hitachi, Tokyo, Japan) were conducted after the steel samples had been polished up to a 1 µm surface finish and etched in a 3% Nital solution. After the macro- and micrographic observations, a Vickers hardness test of the two welded samples was performed with a constant force of 500 gf for 10 s, and the hardness distributions in different zones of the welded joints were obtained.

The HIC sensitivity was evaluated according to the NACE TM0284 standard method [18], and the sensitivity indices of the crack length ratio (CLR (%)) and crack area ratio (CAR (%)) were determined using an ultrasonic detection method. To ensure reproducibility, three samples obtained from the two tested materials (Steel A and B) were evaluated. After the HIC tests, the diffusible hydrogen contents introduced in the samples were measured using a glycerin volumetric method in reference to JIS Z3113 standard [19]. For this, immediately after the HIC tests, the samples were inserted into the glycerin column that was maintained at 45 °C, using liquid nitrogen as a medium to prevent hydrogen diffusion out of the samples. After three days, the volume of hydrogen collected at the top of the glycerin column was measured.

For the mechanistic study, three types of electrochemical measurements (open circuit potential (OCP), potentiodynamic (PD) polarization, and galvanic current) were conducted in a simulated wet sour environment (5% NaCl + 0.5% CH_3_COOH + 0.05 M Na_2_S solution). A typical three-electrode cell composed of a steel sample, a Pt grid, and a saturated calomel electrode (SCE), which acted as the working, counter, and reference electrode, respectively, was used for the OCP and PD polarization measurements. Before the tests, the samples were ground to 2400 grit sand-paper and cleaned ultrasonically in ethanol. For the PD polarization, the potential was scanned from −500 mV to 500 mV vs OCP at a scan rate of 0.2 mV/s. A potentiostat (Gamry reference 600, Pennsylvania, America) in zero-resistance ammeter (ZRA) mode was used to measure the variations in the galvanic current flow between the WM and BM with dimensions of 1 cm^2^. The distance between the two electrodes was 20 mm. With these electrochemical analyses, the surface and cross-section morphologies of the welded steel sample were observed after immersion in a simulated wet sour solution for seven days.

## 3. Results and Discussion

Figure 1 shows the macrostructure, microstructure, and hardness profile of the two welded steel samples. The major differences between the two BMs of the two samples lie in the banding index of pearlite and the level of the 2nd phase particles, which was reported previously [20], but they were not the focus of the present study. Under the same welding conditions and consumables, however, there was no significant difference in the macro- and microstructures of weldments of the two samples. The distributions of the hardness profile of the two welded samples showed a similar pattern: the highest and lowest hardness values were measured around the fusion lines and the BM, respectively.

According to common knowledge in welding metallurgy, a HAZ with high hardness and a coarse grain can be considered the most critical and problematic area in a high-strength steel weld [21,22,23]. For this reason, the SSC of a welded steel sample, which is equivalent to the sample in this investigation, occurred in the HAZ, which has been discussed elsewhere [21,24,25] and is not covered in the present study. The point the authors try to make in this study is to clarify the underlying mechanisms of the changes in the HIC levels with the presence of a narrow weldment in steel samples.

**Figure 2:**
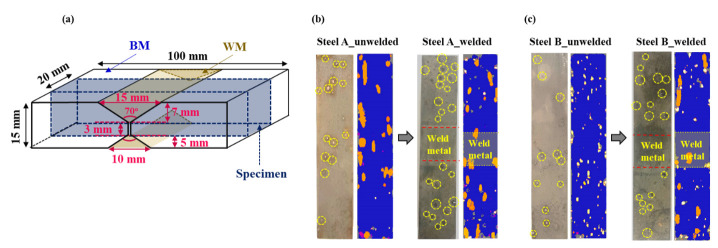
(**a**) Schematic diagram showing the dimensions of welded sample for HIC test in reference to NACE TM0284 standard method; ultrasonically detected HICs of the unwelded and welded samples after HIC test: (**b**) Steel A and (**c**) Steel B.

Figure 2 presents the ultrasonically detected HICs of the unwelded and welded steel samples, which had been immersed in a NACE solution saturated with H_2_S. The differences in the HIC sensitivity (Table 3) between the two unwelded samples are discussed elsewhere [20]. The focus here was on the changes in the HIC levels and distributions after welding the steel samples.

**Figure 3:**
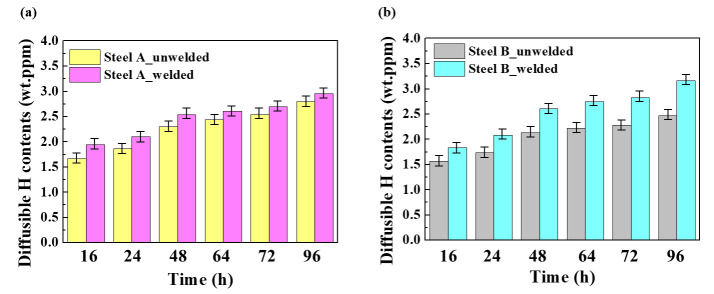
Diffusible hydrogen contents in the unwelded and welded samples: (**a**) Steel A and (**b**) Steel B. (Error bars represent the standard deviations of the mean values).

In contrast to common expectation, most HICs occurred in the BM in the welded samples, not in the HAZ or WM. Moreover, it is interesting to note that the HIC levels in the BM of both welded steel samples were even higher than those of unwelded samples. Under the same materials in the absence of an externally applied stress, the higher susceptibility to HIC could mainly be due to the higher infusion of hydrogen in the materials [26,27]. This suggests that the presence of a weldment in the tested sample could lead to additional hydrogen uptake in the BM some distance from the weldment. This is supported by the increase in the amount of diffusible hydrogen contents ([H]_diff._) introduced in the tested samples after welding, as shown in Figure 3. Although the [H]_diff._ is the sum of the diffusible hydrogen contents obtained from the BM, HAZ, and WM, and each contribution cannot be extracted separately, judging from the HIC occurrence location (Figure 2), most of it may be obtained from the BM.

**Figure 4:**
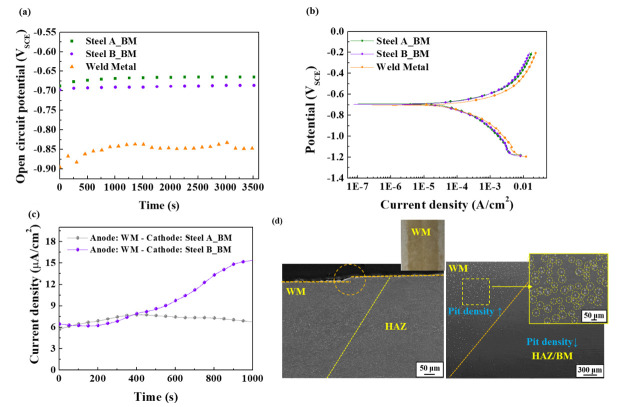
Measurements of (**a**) OCP, (**b**) PD polarization, and (**c**) Galvanic current; (**d**) Metallographic observation of weld metal in Steel A after an immersion test.

An electrochemical approach can be adopted to understand the underlying mechanism behind the higher hydrogen infusion and resulting HIC in the BMs of the welded samples. Because the WM and BM are connected electrically, an electrochemical potential difference can be generated through the differences in chemical composition between them, leading to a galvanic current flow from the anode to the cathode. Figure 4a shows that the WM has a slightly lower open circuit potential than the BM, indicating that the WM may be a more active electrode and act as an anode. Although no significant differences in the PD polarization curves (Figure 4b) between the BM and WM of each steel sample were observed, the anodic reaction (Fe → Fe^2+^ + 2e^−^) and cathodic reaction (H^+^ + e^−^ → H) rates of the WM were slightly higher than those of the BMs. From a practical aspect, the difference in corrosion current density (*i_corr_*) between the WM and BM appears to be insignificant. Nevertheless, there could be sufficient driving force for galvanic corrosion between the WM and BM when they are coupled, which can be supported by the galvanic current flow and current level, as shown in Figure 4c. The measured positive current density also indicates that the galvanic current flows from the WM to the BM, and more electrons can be supplied to the BM. In particular, the geometry of the welded steel sample, or even large-sized welded steel structures such as welded pipes, can provide a large cathode (BM) and small anode (WM) ratio. This ratio can be another significant factor expediting galvanic corrosion. From an electrochemical perspective, the larger the cathode area compared to the anode, the greater the galvanic current, which is the more favorable condition for galvanic interactions. Hence, it can be accepted that the anodic steel dissolution and cathodic hydrogen reduction are dominant on the WM and BM, respectively, in the welded sample. The preferential dissolution and much higher density of localized corrosion damage of the WM in the welded sample, shown in Figure 4d, can be the metallographic observation of galvanic corrosion.

**Figure 5:**
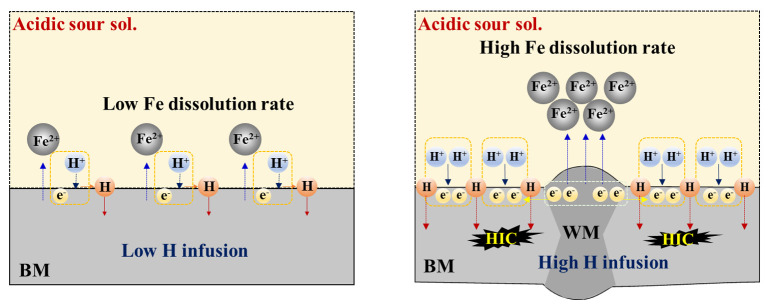
Brief schematic illustration showing the mechanism of galvanic corrosion between the WM and BM, and hydrogen infusion and cracking in the BM.

The formation of this type of galvanic couple between the WM and BM in an acidic sour solution leads to more hydrogen reduction (H^+^ + e^−^ → H) in the BM, resulting in the higher infusion of hydrogen in the BM and more vulnerability to HIC. This process is illustrated schematically in Figure 5.

## 4. Summary

This work elucidates the preferential occurrence of HIC in the BM in the welded steel under wet sour corrosion with a series of experimental results. The major findings are summarized as follows.

The preferential occurrence of HIC in the BM is caused primarily by the fact that the chemical composition of WM, which was optimized for mechanical properties, is slightly anodic to the BM. Even if the WM is close in chemical composition to the BM, the dendritic and inhomogeneous microstructure of the WM can also produce a potential difference with the BM, leading to galvanic corrosion. This leads to the galvanic current flows from the WM to the BM, suggesting that more electrons can be supplied to the BM. Hence, the cathodic hydrogen reduction is more dominant on the BM resulting in the higher infusion of hydrogen in the BM (i.e., welded samples had 5.73% (Steel A) and 28.45% (Steel B) higher [H]_diff._ than unwelded samples) and more susceptibility to HIC (i.e., welded samples had 66.51% (Steel A) and 167.11% (Steel B) higher CLR (%) than unwelded samples). Therefore, optimizing the WM so that its chemical composition is very close to that of the BM or slightly nobler than that of the BM is an effective strategy to suppress preferential anodic dissolution and the formation of HIC in the WM and BM, respectively, in the welded steels under an acidic sour environment.

## Figures and Tables

**Table 1:** Chemical compositions of the two tested steel samples.

Specimens	C	Mn	Si	S	P
Steel A	0.15–0.18	1.1–1.15	0.3–0.4	<0.003	<0.005
Steel B	0.12–0.15	1.1–1.15	0.3–0.4	<0.003	<0.005

**Table 2:** Welding parameters and calculated heat input.

	Electrodes	Welding Current (A)	Arc Voltage (V)	Welding Speed (cm/min)	k-Coefficient	Heat Input (kJ/cm)
Inside	Lead (DC)	650	28	65	1	30.1
	Tail (AC)	480	30			
Outside	Lead (DC)	850	30	85		33.6
	Tail (AC)	650	34			

**Table 3:** Mean values (μ) and their standard deviations (σ) of ultrasonically detected CLR (%) and CAR (%) values of the two samples after HIC test in reference to NACE TM0284 standard method.

Specimens		CLR (%)		CAR (%)	
		μ	σ	μ	σ
Steel A	unwelded	4.48	1.82	7.88	2.04
	welded	7.46	1.98	7.58	2.35
Steel B	unwelded	1.49	0.88	2.24	0.87
	welded	3.98	1.12	5.81	1.86

## Data Availability

The data that support the plots and other findings of the current study are available from the corresponding author on reasonable request.
